# Brain morphology network profiles in male and female youth with autism

**DOI:** 10.1093/cercor/bhag156

**Published:** 2026-09-28

**Authors:** Nooshin Safari, Isaac Levy, Aiying Zhang, Chirag Agarwal, Jesus M Cortes, Kevin A Pelphrey, John Darrell Van Horn, Javier Rasero

**Affiliations:** School of Data Science, University of Virginia, 1919 Ivy Rd, Charlottesville, VA 22903, United States of America; School of Data Science, University of Virginia, 1919 Ivy Rd, Charlottesville, VA 22903, United States of America; School of Data Science, University of Virginia, 1919 Ivy Rd, Charlottesville, VA 22903, United States of America; School of Data Science, University of Virginia, 1919 Ivy Rd, Charlottesville, VA 22903, United States of America; Computational Neuroimaging Laboratory, BioBizkaia Health Research Institute, Plaza Cruces S/N, 48903 Barakaldo, Bizkaia, Spain; Ikerbasque, Basque Foundation for Science, Plaza Euskadi 5, 48009 Bilbao, Bizkaia, Spain; Department of Cell Biology and Histology, University of the Basque Country (UPV/EHU), Barrio Sarriena S/N, 48940 Leioa, Bizkaia, Spain; Department of Neurology, University of Virginia, Ivy Translational Research Building, 560 Ray C Hunt Drive, Charlottesville, VA 22903, United States of America; School of Data Science, University of Virginia, 1919 Ivy Rd, Charlottesville, VA 22903, United States of America; Department of Psychology, University of Virginia, Gilmer Hall, 485 McCormick Rd, Charlottesville, VA 22904, United States of America; School of Data Science, University of Virginia, 1919 Ivy Rd, Charlottesville, VA 22903, United States of America

**Keywords:** autism spectrum disorder, adolescent neuroimaging, morphological networks, multivariate analysis, sex-related profiles

## Abstract

Neuroimaging studies based on altered functional and structural connectivity have contributed to better characterizing males and females with Autism Spectrum Disorder (ASD), advancing our understanding of the male prevalence in diagnosis. However, much less is known about how brain networks are altered from a morphological perspective, and whether these alterations help explain sex-related characteristics in ASD. Here, we used structural MRI from a sex- and diagnosis-balanced sample of 337 young individuals (8 to 18 years) from the Autism Center of Excellence to elucidate sex-related alterations in morphology-based connectivity, calculated as the similarity between region-wise multivariate morphological signatures. Although no diagnosis-by-sex interaction network profile was found, within-sex analysis demonstrated that in males, ASD had significantly increased morphology-based connectivity involving the fusiform gyrus, medial orbitofrontal, entorhinal, and parahippocampal cortices. This network profile was linked to a core social–communication trait within the autistic group. Females with ASD exhibited a network profile notably comprising increased morphology-based connectivity in the entorhinal cortex, followed by the inferior parietal lobule and lateral occipital cortex; yet it lacked association with core autistic traits. These within-sex profiles had minimal overlap with networks from normative sex differences. Together, these findings offer new insights into the neurobiology of males and females with autism.

## Introduction

Autism Spectrum Disorder (ASD) is a neurodevelopmental condition defined by persistent impairments in social communication alongside restricted and repetitive behaviors, currently affecting approximately one in 36 children in the United States, with a male-to-female diagnosis ratio ranging from 3:1 to 4:1 (Loomes et al. 2017; Baio et al. 2018). The basis of this sex bias is still under debate. Some theories suggest that ASD is characterized by a cognitive trait dominated by systemizing rather than empathizing. Systemizing is more commonly observed in males, which may explain the higher prevalence of autism in this sex. Under this view, females are at the opposite end of the cognitive profile due to their generally stronger ability for empathizing (Baron-Cohen 2002). Another compelling theory is the Female Protective Effect, which posits that females are more resilient to developing autism and require a greater etiological load to reach diagnosis (Robinson et al. 2013). If these theories are true, they should be at least partially reflected in the brain through sex-specific neural correlates. To examine this, sex should be treated as a stratifying variable rather than a nuisance covariate (Halladay et al. 2015; Lai et al. 2015).

Much of our understanding of the neurobiology underlying males and females with autism has emerged from neuroimaging efforts evaluating disruptions in brain circuitry. From a functional connectivity perspective, typically assessed as the similarity in blood-oxygen-level dependent (BOLD) activity between brain areas, females with ASD typically show a trend toward network-wide hyperconnectivity relative to sex-matched typically developing controls (TDCs), while males tend toward hypoconnectivity (Alaerts et al. 2016). At a more local level, these patterns can be more nuanced or even reversed (Di and Biswal 2016). In particular, functional disruptions in females with ASD are predominantly found in somatomotor regions, while males exhibit alterations in both the default mode and somatomotor networks—supporting the so-called Gender Incoherence model (Floris et al. 2018). Additional differences in visual, language, and basal ganglia connectivity have also been reported among autistic females (Tavares et al. 2021). Similar sex-related reports have been found in structural connectivity, typically quantified using diffusion MRI to extract multiple properties in white matter tracts connecting brain regions. In preschool-aged children, males with ASD show reduced callosal projections to the orbitofrontal cortex, whereas females exhibit reductions targeting anterior frontal areas, along with elevated diffusivity metrics (Nordahl et al. 2015). During adolescence, females with ASD—but not males—show reduced fiber density and cross-section in callosal pathways (Kirkovski et al. 2020). Region-specific reductions in connectivity involving temporal, parietal, and medial posterior cortices have also been reported in females, with no corresponding effects in males (Irimia et al. 2017).

Brain morphology has also contributed to further characterizing sex-specific differences in autism. Recent findings indicate that autistic girls exhibit thicker cortices in early development, followed by accelerated thinning relative to autistic boys, suggesting a sex-linked protective mechanism (Andrews et al. 2024). Overall, morphological features (e.g., cortical thickness, surface area, sulcal depth, mean curvature, and volume) are thought to reflect the cumulative outcome of developmental and genetic processes, positioning them as important markers for disentangling heterogeneity in ASD (Yang et al. 2016). Yet, most of this work has focused on region-by-region analyses, typically examining features independently across predefined brain areas, which may be insufficient to capture the spatial interdependencies in brain morphology that sustain neurodevelopment (Bedford et al. 2019).

One promising approach to overcome this limitation is to also adopt a connectivity perspective on brain morphology by examining the similarity in patterns of morphometric information (e.g., cortical thickness, surface area) between brain regions. Most efforts in this direction have employed structural covariance networks, which model connectivity as inter-regional covariation in a single morphometric property across individuals (Tijms et al. 2011; Kong et al. 2015). In the context of autism, studies have reported widespread alterations in connectivity based on region-wise volumetric covariation, with some of these patterns linked to socio-cognitive differences (Cardon et al. 2017; He et al. 2021). A recent study further identified significant sex-by-diagnosis interactions in this type of network within major brain systems, showing that ASD males had increased covariance relative to typically developing males, while ASD females displayed reduced covariance compared to female controls (Zielinski et al. 2022). Despite these findings, the characterization of males and females with autism through alterations in morphology-based connectivity remains largely underexplored. Moreover, common structural covariation network approaches do not leverage all the morphological information in the brain as they are typically limited to a single property, which may overlook the benefits of integrating multiple features into a unified framework to provide a more comprehensive description of group and individual differences in cortical organization (Li et al. 2017; Seidlitz et al. 2018).

To fill these gaps, the present study set out to better understand ASD in males and females during typical developmental ages by characterizing network profiles using measures of connectivity defined from spatial patterns of multiple morphological properties together. To this end, we leveraged a large-scale, multi-site dataset comprising 337 youth participants (aged 8 to 18 years), balanced by sex (162 females) and diagnosis (181 with ASD). Adopting a novel framework for robustly estimating similarity networks from T1-weighted images (Sebenius et al. 2023), morphology-based connectivity was computed by measuring the similarity between brain regions in their vertex-wise multivariate distributions of morphological features, including thickness, mean curvature, surface area, sulcal depth, and gray matter volume. Clusters of significantly disrupted connections in ASD relative to TDC were then examined for both within males and females separately, and for between sexes (i.e., sex-by-diagnosis interactions). In addition, the associations of identified clusters with behavioral and comorbidity traits were further evaluated.

## Materials and methods

### Participants

337 individuals from Wave 1 of NIH Autism Centers of Excellence (ACE) Network were selected for this study after quality control assurance, involving visual inspection of segmentation output and exclusion of scans with structural preprocessing failures. The ACE network specifically focused on investigating sex differences in children with ASD by recruiting equal numbers of females and males diagnosed with autism (see Jacokes et al. 2025 for more details). The study sample included 181 subjects with ASD (Age: 12.73 ± 2.92 years, 84 female, 136 identifying as White, 8 identifying as Black, 4 identifying as Asian, 28 identifying as multiracial, 2 reporting other racial identities, 3 reporting no racial identity, 147 identifying as not Hispanic, 31 identifying as Hispanic, and 3 reporting no ethnic identity) and 156 with TDC (age: 13.20 ± 3.01 years, 78 female, 115 identifying as White, 13 identifying as Black, 11 identifying as Asian, 16 identifying as multiracial, 1 reporting no racial identity, 117 identifying as not Hispanic, 36 identifying as Hispanic, and 3 reporting no ethnic identity), who were collected across four sites: Harvard University, Seattle Children’s/University of Washington, University of California Los Angeles, and Yale University (Fig. 1A). No statistical group differences in site proportions were observed across sex strata (Cochran–Mantel–Haenszel test *M*^2^ = 0.83, *df* = 3, *P* = 0.84) and within sexes (males: *χ^2^* = 3.86, *df* = 3, *P* = 0.28; Females: *χ^2^* = 0.93, *df* = 3, *P* = 0.82). Additionally, several scores were used to assess the association of the morphology-based connectivity within the identified network profiles with behavioral and comorbidity traits in individuals with autism. Specifically, we extracted scores for communication, social interaction, and restricted, repetitive, and stereotyped behaviors from the Autism Diagnostic Interview–Revised (ADI-R); social affect and restricted and repetitive behaviors from the Autism Diagnostic Observation Schedule—2nd Edition (ADOS-2); and subscale scores for anxiety, affective problems, attention deficit/hyperactivity, oppositional defiant, conduct, and internalizing problems from the Child Behavior Checklist (CBCL). Descriptive statistics for our sample can be found in Table 1.

**Figure 1:**
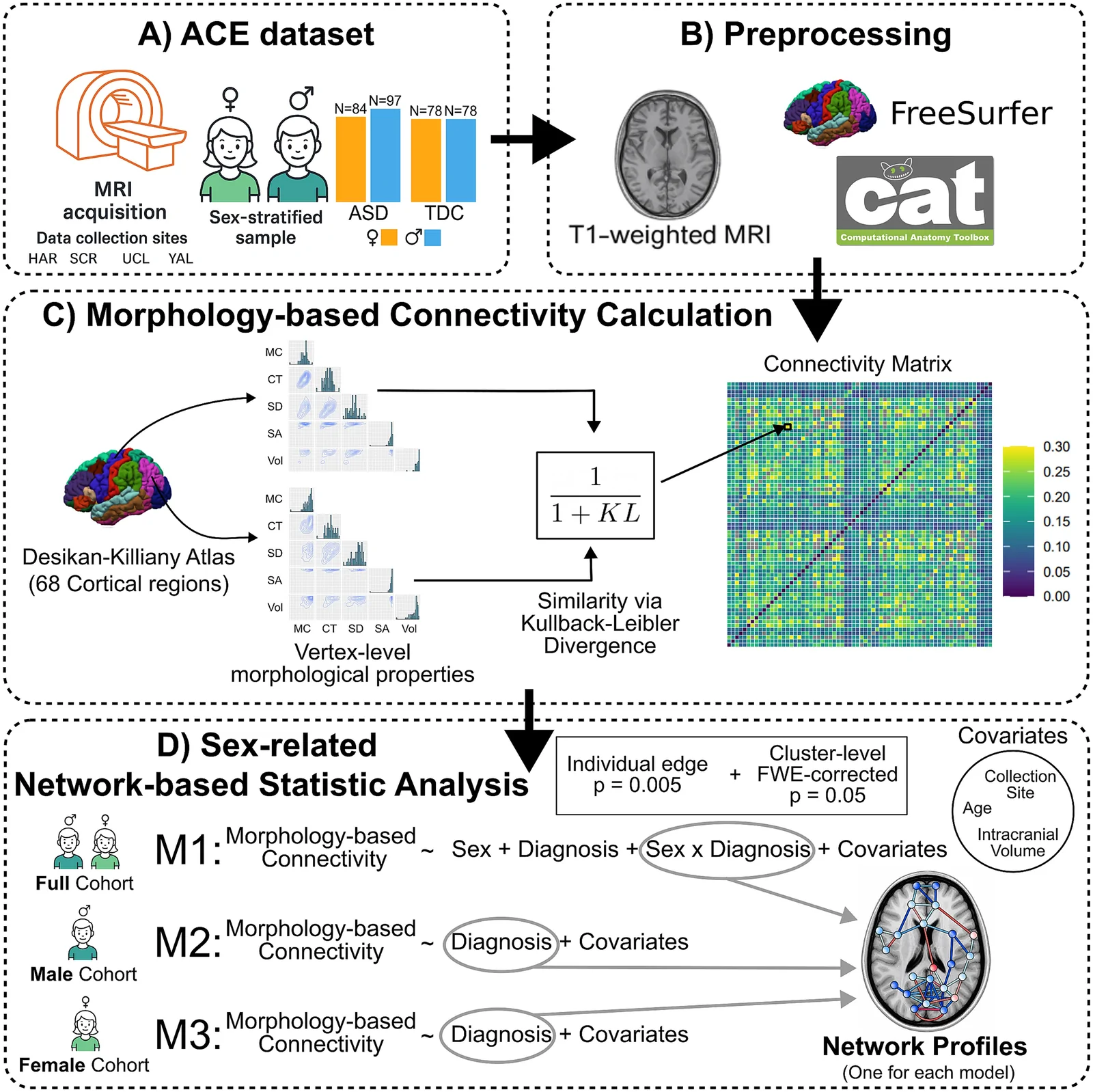
Methodological workflow. A) We analyzed a sex- and diagnosis-balanced sample of 337 individuals aged 8 to 18 years. B) T1w images were preprocessed using FreeSurfer and CAT12 for voxel-based morphometry. C) From the preprocessed data, we extracted regional distributions of multivariate morphological features (mean curvature, cortical thickness, sulcus depth, surface area, and volume) defined by the Desikan–Killiany cortical atlas. Inter-regional morphology-based connectivity was then estimated using the Kullback–Leibler divergence between these multivariate distributions. D) Finally, a network-based statistic was used to find network profiles representing clusters of connections significantly associated with sex-by-diagnosis effects in the full sample (M1), and with ASD-TDC differences within each sex (M2 and M3). Across these models, age, intracranial volume and collection site were included as covariates.

**Table 1:** Demographic, behavioral and comorbidity data stratified by sex and diagnostic group.

	Male		Female	
Variable	ASD	TDC	ASD	TDC
N	97	78	84	78
Age (months)	152.36 [145.02–159.69]	161.02 [153.47–168.57]	153.34 [146.03–160.65]	155.82 [147.10–164.55]
Site (HAR/SCR/UCL/YAL)	24/28/30/15	11/23/26/18	11/25/25/23	13/25/23/17
ICV (${10}^6\times$ mm${}^3$)	1.63 [1.60–1.66]	1.64 [1.61–1.67]	1.47 [1.44–1.50]	1.48 [1.45–1.51]
ADI-R Social	19.38 [18.28–20.49]	—	18.11 [16.74–19.48]	—
ADI-R Communication	16.54 [15.67–17.42]	—	15.38 [14.37–16.40]	—
ADI-R Behavioral	6.63 [6.12–7.14]	—	5.70 [5.13–6.28]	—
ADOS-2 Social Affect	9.52 [8.75–10.28]	—	8.06 [7.40–8.72]	—
ADOS-2 Communication	2.63 [2.29–2.97]	—	2.54 [2.15–2.93]	—
CBCL Affective	61.07 [59.28–62.85]	51.60 [50.86–52.33]	64.19 [61.93–66.46]	52.95 [51.87–54.02]
CBCL Anxiety	61.74 [60.13–63.36]	51.90 [51.06–52.73]	62.49 [60.56–64.43]	52.04 [51.14–52.94]
CBCL ADHD	61.92 [60.37–63.48]	50.51 [49.00–52.01]	63.38 [61.47–65.28]	51.18 [50.59–51.77]
CBCL Oppositional	58.65 [57.04–60.26]	51.62 [50.96–52.28]	58.38 [56.67–60.09]	51.53 [50.94–52.13]
CBCL Conduct	56.17 [54.70–57.63]	50.81 [50.32–51.29]	57.33 [55.56–59.10]	50.86 [50.38–51.35]
CBCL Internalizing	60.87 [59.15–62.58]	43.81 [41.66–45.95]	63.04 [60.48–65.60]	46.47 [44.31–48.63]

Values represent means and 95% confidence intervals. Dashes (—) indicate unavailable scores for TDC participants. HAR: Harvard University; SCR: Seattle Children’s/University of Washington; ICV: intracranial volume.

### MRI acquisition and preprocessing

Structural images were acquired using a high-resolution T1-weighted MPRAGE sequence (FOV: 176*×*256*×*256; voxel size: 1*×*1*×*1 mm^3^). Preprocessing of these images was performed with FreeSurfer’s recon-all pipeline (v6.0), which involves cortical surface reconstruction and subcortical segmentation. After completion, this pipeline provided estimates of the specific brain morphological properties at each cortical vertex, which were subsequently used to compute the individual-level connectivity matrices (see next sub-section). Additionally, gray matter volume maps were generated using a standard voxel-based morphometry pipeline implemented in CAT12 (Gaser et al. 2024), and transformed into the FreeSurfer subject space (Fig. 1B). These maps enabled the extraction of voxel-wise gray matter distributions for cortical and subcortical regions, needed to compute whole-brain morphology-based connectivity matrices (see next sub-section). This included spatial normalization, modulation, and smoothing of gray matter probability maps. On average, T1w images exhibited satisfactory quality (*78.07± 5.28*), as quantified using the weighted Image Quality Rating (IQR) metric, which combines contrast noise, motion-related bias and resolution information (Gaser et al. 2024). IQR values were used to further test the robustness of the observed results to image quality and motion-related noise.

### Morphology-based connectivity

Morphology-based connectivity matrices were constructed for all subjects using the Morphometric Inverse Divergence (MIND) framework (Sebenius et al. 2023), which estimates pairwise similarity between brain regions based on the symmetrized Kullback–Leibler (KL) divergence of vertex-wise morphometric feature distributions (Fig. 1C). Therefore, morphology-based connectivity was defined using statistical dependencies between regions as typically done in functional connectivity, but quantifying their similarity based on spatial patterns of morphological information instead of on temporal profiles of BOLD signals. Moreover, in contrast to traditional structural covariance network approaches, this novel framework enabled the integration of multiple morphological brain properties (e.g., thickness, volume) into one compact connectivity matrix. It also yielded more reliable individual connectivity estimates, as such integration was done at the vertex level, in contrast to existing multivariate approaches that use arrays of standardized summary statistics (one per feature) at the region level, which unrealistically force each MRI feature to be equally variable across cortical areas (Sebenius et al. 2023). Main analyses concentrated on connectivity between cortical regions, where multiple morphological properties could be defined. Additional analyses were also performed at the whole-brain, but these were based solely on volumetric information, since this was the only morphological measure available for subcortical regions.

Specifically, to build the morphology-based connectivity matrices at the cortical level, the vertex-level outputs for thickness, mean curvature, surface area, sulcal depth, and gray matter volume, as estimated by FreeSurfer, were first extracted for each region of the Desikan–Killiany atlas (68 regions of interest, ROI; 34 per hemisphere). Then, for each region, these feature-specific data were combined and their multivariate distribution modeled using a kernel density estimator based on k-dimensional trees. Finally, a symmetrized multivariate KL divergence was employed to compute the similarity between pairs of regions (see Sebenius et al. 2023 for the exact mathematical details). This resulted in a 68 × 68 connectivity matrix for each individual, representing the similarity between regions based on their multivariate morphological signatures.

For the whole-brain morphology-based connectivity matrices, 14 subcortical structures obtained from FreeSurfer’s *aseg.mgz* segmentation file (Fischl 2012) were added to the aforementioned cortical regions from the Desikan–Killiany atlas. These subcortical regions consisted of the bilateral thalamus, caudate, putamen, pallidum, hippocampus, amygdala, and nucleus accumbens. An 82 × 82 connectivity matrix was then built for each individual as the univariate KL divergence between region-wise gray matter volume distributions, extracted from the voxel values within each ROI after the voxel-based morphometry pipeline.

### Network profiles

Network-Based Statistic (NBS) (Zalesky et al. 2010) was used to identify network profiles as connected components (“clusters”) of statistical differences in morphology-based connectivity. NBS is analogous to cluster-wise thresholding of statistical brain maps but adapted for network data (Fig. 1D). First, for each edge in the connectivity matrix, a multiple linear regression model was fitted across participants to estimate its two-sided association with an effect of interest while controlling for covariates. Three statistical models were separately considered, each intended to provide a different sex-related network characterization: M1) a sex-by-diagnosis interaction effect, M2) ASD vs. TDC in males and M3) ASD vs. TDC in females. For M1, the sex × diagnosis interaction term was tested in the full cohort, with sex and diagnosis included as main effects. For M2 and M3, diagnosis was tested within each sex separately. Across these three statistical models, covariates comprised age, intracranial volume, and collection site. By including intracranial volume as a covariate, we helped ensure that tested effects reflected connectivity differences rather than global volumetric scaling.

After statistical estimation, an individual-edge *P*-value associated with the effect of interest was calculated. A threshold, *p**,* was then applied to these *P*-values to identify suprathreshold edges, which were assembled into connected components or clusters, each with a size equal to the number of suprathreshold edges and an intensity equal to the sum of the associated edge statistics. In NBS, this threshold is a hyperparameter that determines the potential size of connected components or clusters. We chose *p=0.005* since it provided a compromise in cluster size, sufficient for discovery while preserving statistical power (Zalesky et al. 2010). To assess robustness, we also tested the sensitivity of the findings to a more liberal (*p=0.01*) and stringent threshold (*p=0.001*).

Finally, the significance of each connected component or cluster was assessed using permutation testing while controlling the family-wise error rate. This was done through a maximum-statistic inference approach. Specifically, in each permutation, the original data were randomly permuted, and the same edge-level statistical estimation, thresholding, and component-identification procedures were repeated. Null distributions for component size and intensity were empirically estimated by retaining their respective maximum values from each permutation. The present study used 5,000 permutations and applied a standard family-wise error threshold of ${P}_{FWE}=0.05$ to identify significant network profiles.

This analysis was implemented using the *nbr_lm* function from the NBR R package (Gracia-Tabuenca and Alcauter 2020).

### Importance of morphological properties

The relative importance of each morphological property in distinguishing individuals with autism from TDCs was assessed by combining the strength of each sub-network identified by NBS with a leave-one-feature-out procedure within an ROC curve analysis framework. Strength was calculated for each individual’s morphology-based connectivity matrix as the sum of values corresponding to the altered sub-networks. This metric reflects the overall magnitude of morphological similarity within the significant sub-networks and provides a compact summary of network-level disruption. The leave-one-feature-out procedure removed a given morphological property prior to constructing the connectivity matrices via multivariate KL divergence, thereby changing the strength of the sub-networks too. The impact on classification performance was then quantified by examining the change in the area under the ROC curve (AUC) using strength as the discriminating variable. This procedure was repeated for each morphological property individually.

### Association with behavior and comorbidity

Within the autistic group, we applied Partial Least Squares (PLS) regression to test the association between the significantly altered morphology-based connectivity edges identified by NBS and multivariate behavioral information, comprising the social, communication, and behavioral subscores from ADI-R, as well as the behavioral and social affect scores from ADOS-2. PLS aims to find latent components such that a multivariate dataset *X* (here, connectivity values of significantly altered edges by NBS) maximally covaries with another multivariate dataset *Y* (here, multivariate behavioral information). Null distributions for each latent component were generated by repeatedly running PLS with the observation-wise permutation of *Y* (5,000 permutations), and statistical significance was claimed at the 0.05 threshold after FDR correction. Furthermore, contributions of individual features in *X* and *Y* to each significant latent component were assessed by inspecting the corresponding loading profiles, focusing on those features whose loadings significantly deviated from zero at a 95% confidence interval calculated by bootstrapping (5,000 iterations). This analysis was implemented using the *pyls* Python package (https://pyls.readthedocs.io/en/latest). To maintain consistency with the covariates used in the primary NBS analysis, the effects of age, site, and intracranial volume were regressed out from both connectivity- and behavior-based datasets before the PLS regression.

The same approach was adopted to assess the relationship between the significantly altered connectivity edges and comorbidity symptoms, consisting of affective problems, anxiety problems, attention deficit/hyperactivity, oppositional defiant problems, conduct problems, and internalizing problems scores in CBCL.

### Sensitivity analysis

Several post-hoc sensitivity analyses were performed to test the robustness of the networks identified by NBS, again using their morphology-based connectivity strength as a compact summary of these network-level alterations. First, residual site effects were assessed by evaluating if ASD–TDC differences in connectivity strength of the network profiles varied across acquisition sites. This was done through an ANOVA by testing whether adding a diagnosis-by-site interaction term significantly improved model fit. Second, group differences were tested after including IQR as an additional covariate to assess whether observed results remained significant after accounting for individual differences in image quality.

## Results

### Sex-by-diagnosis interaction effect

NBS identified no significant clusters of altered connections for a sex-by-diagnosis interaction effect across the three different individual edge thresholds, suggesting that the current data did not provide sufficient evidence for a diagnosis-related morphological network profile differing between males and females.

### ASD males vs. TDC males

Group-level analysis identified a single cluster of 35 significantly altered edges (${P}_{FWE}=0.037$). The connectivity strength of this network profile significantly differed between groups (*t* = −5.92, *df* = 168, *P* < 0.001). This group difference did not show evidence of residual site effects (*F* = 0.96, *df_1_* = 3, *df_2_* = 165, *P* = 0.41) and remained significant after accounting for individual variation in image quality (*t* = −4.92, *df* = 167, *P* < 0.001).

As shown in Fig. 2A, the identified network profile mostly corresponded to increased morphology-based connectivity in ASD relative to TDC, with a pattern prominently involving the left hemisphere fusiform and medial orbital frontal gyri, as well as the right hemisphere entorhinal cortex and lingual gyrus. This profile also included a single weak connection showing decreased connectivity in ASD compared to TDC, linking the left middle temporal gyrus and the right inferior temporal gyrus. The prominent role of the fusiform in this network profile can also be seen in Fig. 2B, which depicts the number of significantly altered connections per region projected onto the brain. This analysis was performed using the individual edge threshold *p=0.005* and 5,000 permutations for null modeling. Additional profiles with other individual edge thresholds in NBS were also reported (see Supplementary Fig. S1). Remarkably, the fusiform continued to exhibit significantly increased connectivity even under the most stringent threshold (*p=0.001*). No significant clusters at the whole-brain level using volumetric information only were found at any of the thresholds.

**Figure 2:**
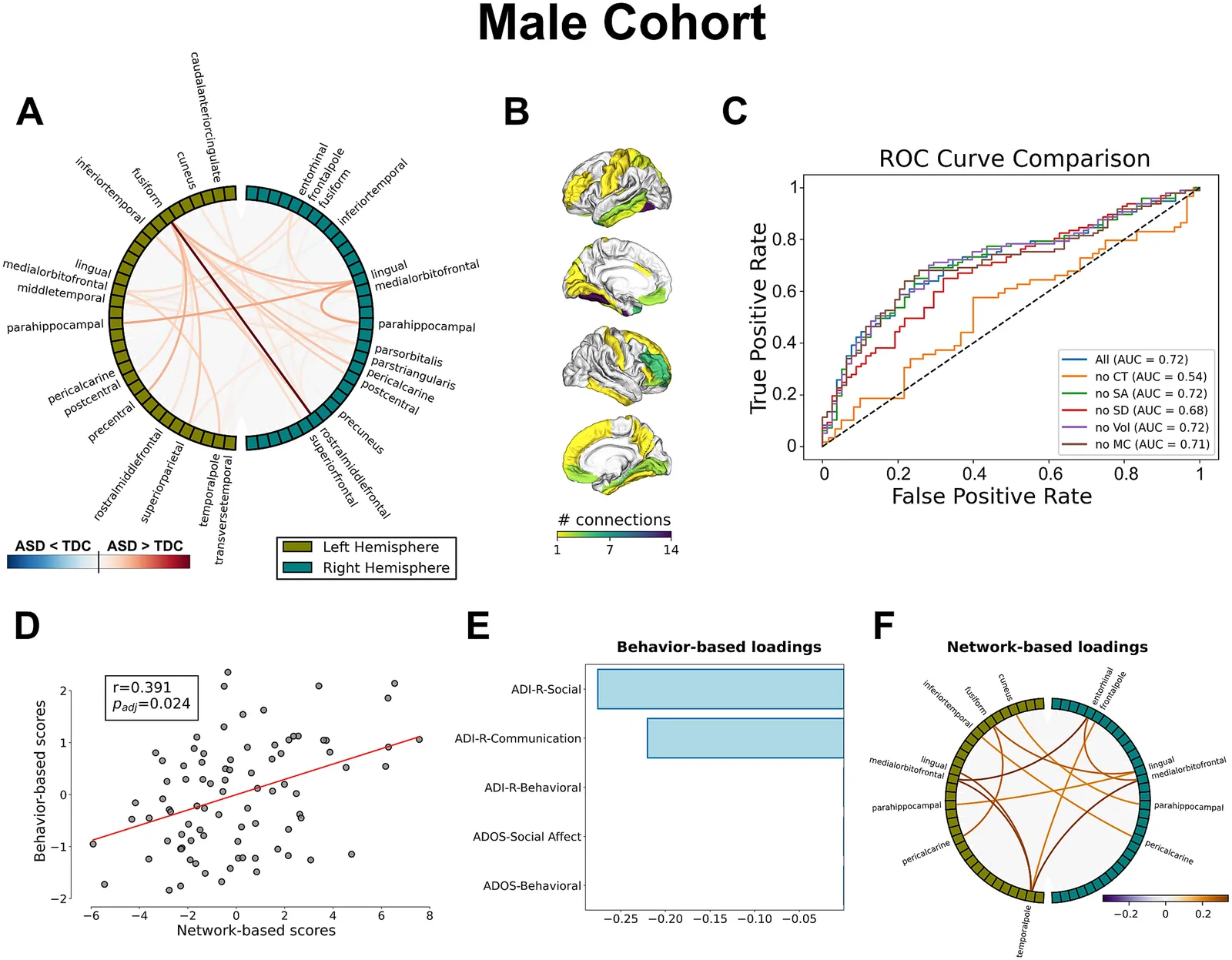
Main results in the male cohort. A) Circular plots visualizing clusters of significant inter-regional connections. Nodes represent cortical regions for each hemisphere. Cool (warm) colors indicate increased (decreased) morphology-based connectivity in TDC relative to ASD. Darker colors denote stronger group differences. B) Brainplot displaying the number of significantly altered connections per region C) ROC curves and area under the curve (AUC) values showing performance in distinguishing ASD from TDC using the strength of network profiles in A. Each curve represents the scenario in which one morphological property is omitted in the computation of the connectivity matrices (CT: cortical thickness, SA: surface area, SD: sulcus depth, Vol: volume, MC: mean curvature). D) Association between morphology-based connectivity and behavior in ASD provided by a significant latent component using Partial Least Squares regression, and their corresponding loadings (E and F panels respectively), focusing only on those contributing factors whose weights are away from zero at a 95% confidence level.

The most influential morphological property for distinguishing between controls and individuals with autism within this cluster appeared to be cortical thickness, with the AUC dropping from 0.72 (when all features were included) to 0.54 when this feature was removed (see Fig. 2C). This was followed by sulcal depth (AUC = 0.68) and mean curvature, although in the latter case the reduction in group separability was minimal (AUC = 0.71). In contrast, surface area and volumetric properties did not appear to be individually necessary, as removing either of them resulted in slightly improved group separability (AUC = 0.72 in both cases).

We found a latent component by PLS (*r* = 0.391, *P_adj_* = 0.024) in which altered morphological connections significantly associate with behavior within the autistic group (Fig. 2D). This association was primarily driven by negative loadings of social and communication scores from ADI-R (Fig. 2E) and positive loadings of the left hemisphere fusiform and temporal pole regions, as well as the right hemisphere entorhinal and lingual cortices (Fig. 2F), indicating a negative correlation between ASD-relevant behavior and altered morphology-based connectivity.

No significant latent components between altered sub-networks and comorbidity information were found after FDR correction.

### ASD females vs. TDC females

Within the female subgroup, a single cluster comprising 41 significantly altered morphology-based connectivity values (${P}_{FWE}=0.028$) was identified. The connectivity strength of this specific network significantly differed between groups (*t* = −3.98, *df* = 155, *P* < 0.001). This group difference did not show evidence of residual site effects (*F* = 0.91, *df_1_* = 3, *df_2_* = 152, *P* = 0.44) and remained significant after accounting for individual variation in motion-related image quality (*t* = −3.25, *df* = 153, *P* = 0.001).

As displayed in Fig. 3A, compared to TDC, patterns of increased connectivity in the ASD group were centered around a prominent hub in the bilateral entorhinal cortex, which then extended to the bilateral inferior parietal lobule, lateral occipital cortex, and pre- and postcentral gyri, all regions implicated in visuospatial integration, somatosensory encoding, and mnemonic retrieval. In contrast, decreased connectivity, although more scarce, prominently involved the bank of the superior temporal sulcus engaging with a few other regions, among which the fusiform gyrus was found. Figure 3B visually aids in highlighting the prominent role of the entorhinal cortex by displaying the number of significantly altered connections per region on the brain. All these alterations remained consistent—yet spatially circumscribed, more notoriously to the left hemisphere entorhinal cortex—at the more stringent individual edge threshold in NBS (*p=0.001*). At the more liberal *p=0.01* level, the pattern expanded to include bilateral precentral extensions and deeper parietal-occipital couplings (see Supplementary Fig. S2), suggesting a core-periphery organization sensitive to statistical stringency. Similar to males, no significantly altered networks were found at any of the thresholds at the whole-brain level using only volumetric information.

**Figure 3:**
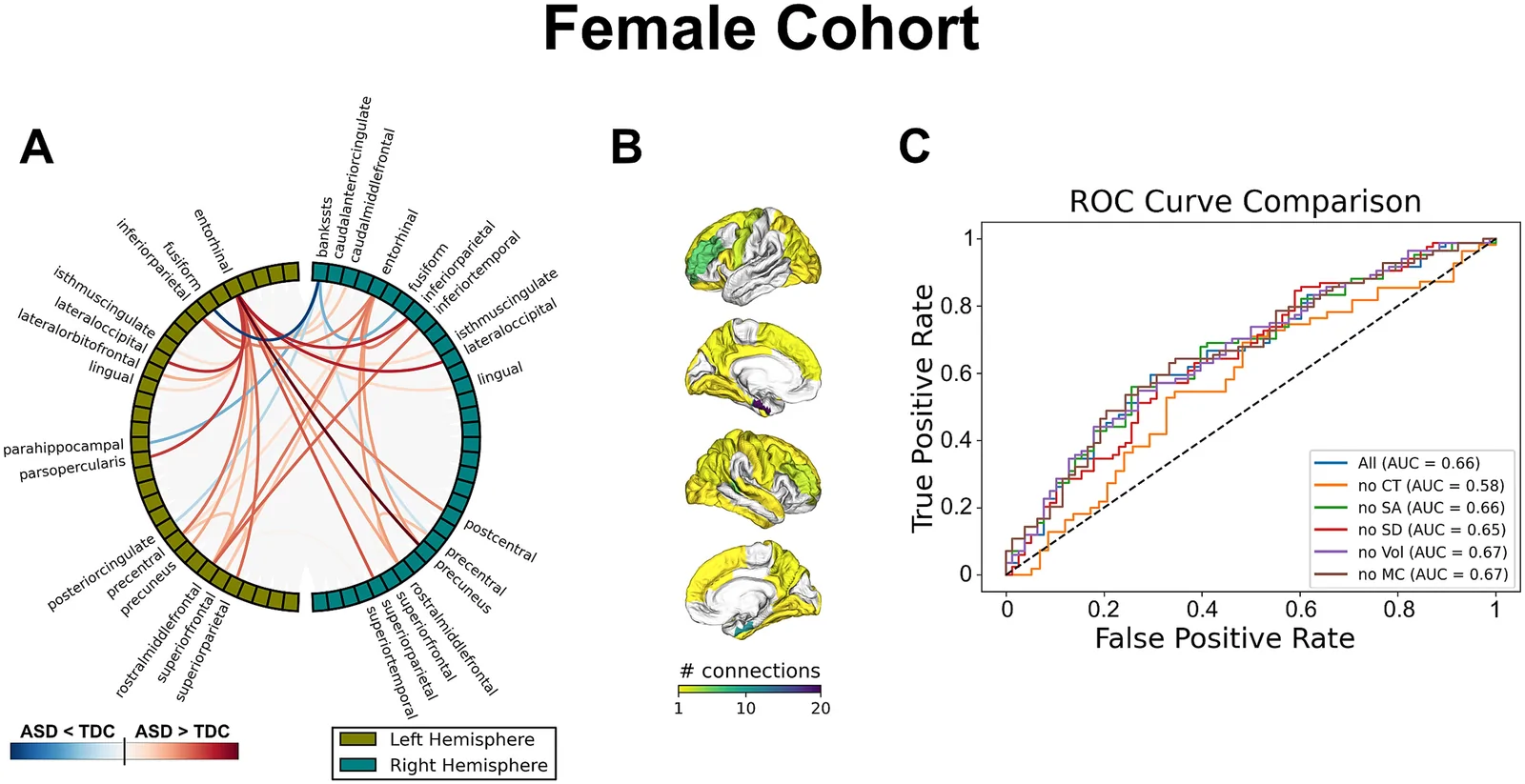
Main results in the female cohort. A) Circular plots visualizing clusters of significant inter-regional connections. Nodes represent cortical regions for each hemisphere. Cool (warm) colors indicate increased (decreased) morphology-based connectivity in TDC relative to ASD. B) Brainplot displaying the number of significantly altered connections per region C) ROC curves and area under the curve (AUC) values showing performance in distinguishing ASD from TDC using the strength of network profiles in A. Each curve represents the scenario in which one morphological property is omitted in the computation of the connectivity matrices (CT: cortical thickness, SA: surface area, SD: sulcus depth, Vol: volume, MC: mean curvature).

In terms of group separability, a similar pattern to that observed in the male cohort was found, to a large extent (see Fig. 3C). Using all morphological features together yielded an AUC of 0.66. Removing cortical thickness led to the largest reduction in group separability (AUC = 0.58), confirming its key role. In contrast, removing surface area had no effect (AUC = 0.66), while sulcal depth had only a minimal impact in this case (AUC = 0.65). Volumetric properties and mean curvature individually did not appear to be essential, as their removal slightly increased the AUC to 0.67 in both cases.

No significant latent components linking altered morphology-based connectivity with either behavioral or comorbidity information in females with autism were found after FDR correction.

### Overlap with normative sex differences

To evaluate whether observed within-sex network profiles could arise from normative sex differences, we applied the same NBS setup of our main analysis (individual edge threshold *p=0.005*, cluster-level family-wise error correction ${P}_{FWE}=0.05$, 5,000 permutations, and identical covariates) to compare TDC males with TDC females. This analysis revealed a connected component of 48 significantly altered edges, which are depicted by directionality in Supplementary Fig. S3. When overlapping this normative sex-difference network with the diagnostic-related profiles, we found a Jaccard index of *J=0* for ASD males vs. TDC males, and *J=0.01* for ASD females vs. TDC females. In the latter case, only a single overlapping connection was found, linking the left rostral middle frontal gyrus and the right superior temporal gyrus. These minimal overlaps indicate that the identified connections within each sex were largely distinct from those arising from normative sex differences.

## Discussion

In this study, we leveraged a large, sex- and diagnosis-balanced cohort of subjects to delineate sex-related profiles of altered brain connectivity derived from multivariate morphometric signatures in young individuals with autism. Although our data did not provide sufficient evidence for a sex-by-diagnosis morphological network profile, when testing for within sex connectivity alterations, males with autism showed a network profile involving heightened levels of connectivity in prominent regions such as the fusiform and lingual gyri, anterior cingulate cortex, orbitofrontal cortex, and parietal association cortices. Furthermore, the morphology-based connectivity values of this specific network were linked with autistic traits in social-communication. Likewise, females were characterized by a network profile exhibiting elevated connectivity in the entorhinal cortex, inferior parietal lobule, and lateral occipital cortex, along with decreased connectivity between the fusiform gyrus and the bank of superior temporal sulcus. No association between their measured autistic traits and morphology-based connectivity values for this specific network was found. Observed network profiles in both females and males had minimal overlap with network profiles from normative sex differences. Together, these findings improve the characterization and understanding of ASD in male and female youth through connectivity measures derived from integrating multivariate morphological information.

Our results add to the growing body of evidence reinforcing a network-level perspective of ASD by explicitly focusing on sex-related characterization and brain connectivity derived from morphological information (Sebenius et al. 2023). For the former, we leveraged data from the ACE network which, in contrast to similar large-scale efforts for the study of autism (Di Martino et al. 2014; Di Martino et al. 2017), was particularly designed to recruit an equal number of males and females with autism. For the latter, we calculated connectivity through MIND, a novel framework for robustly estimating individual-level morphology-based connectivity which, to the best of our knowledge, had not previously been applied to better understand autism in males and females. Prior efforts have also investigated morphology-based networks in autism, providing important and equally valuable insights (Bethlehem et al. 2017; He et al. 2021; Guo et al. 2023; Wang et al. 2024; Jiang et al. 2026). However, these (typically male-biased) studies have traditionally relied on inter-regional covariation in a single morphological property across individuals (structural covariance networks), or, when integrating multiple morphological properties into individual-level connectivity measures (e.g., Morphometric Similarity Networks), have been subject to methodological limitations (see Sebenius et al. 2023 for more details).

In males, one of the central hubs we identified was the fusiform gyrus, a region implicated in social cognition that has been consistently linked to autism through findings of reduced activation during face processing and structural abnormalities associated with social perceptual deficits (Hadjikhani et al. 2004; Pierce and Redcay 2008; Floris et al. 2025). Other key regions included the parahippocampal gyrus (linked to memory) and the postcentral gyrus (involved in sensorimotor processing). These findings align with theories of network-level developmental plasticity, in which increased long-range integration may reflect delayed synaptic pruning or compensatory adaptations to altered sensory or cognitive demands, as supported by studies using graph-theoretical metrics (Zielinski et al. 2010; Rudie et al. 2013). These areas have also been reported as hyperconnected in fMRI studies of male-predominant cohorts (Abbott et al. 2015; Abbott et al. 2017; Keown et al. 2017), contribute to the functional connectivity–based heterogeneity observed in autism (Rasero et al. 2023), and are implicated in disrupted white matter architecture characterized by reduced fractional anisotropy and aberrant streamline coherence along the cingulum bundle, forceps minor, and superior longitudinal fasciculus (Shukla et al. 2011; Travers et al. 2012; Aoki et al. 2017). Overall, our findings point to a coherent pattern of multimodal dysconnectivity in males with ASD, centered on frontal, temporal, and occipital integration hubs as key components of ASD neuropathophysiology.

On the other hand, in females with ASD, key regions identified here align with prior findings from both functional and structural networks. For example, resting-state fMRI studies have revealed disruptions within the default-mode network (heightened entorhinal–posterior cingulate), altered top-down central executive influence—specifically dorsolateral prefrontal to postcentral pathways—and increased coupling between the default-mode and central executive networks (Just et al. 2012; Hong et al. 2019). Interpreted within the canonical triple-network model (Menon 2011), these changes suggest a reconfigured network topology in ASD females, which may reflect compensatory upregulation of executive–memory loops to offset local cortical variability. Additionally, sex-specific salience network alterations—such as elevated connectivity with sensorimotor areas reported in early ASD trajectories (infants at familial risk) (Tsang et al. 2024)—may reflect dynamic sensory reweighting and flexible attention gating across internal–external stimulus axes. Further, reduced network flexibility in executive and salience systems (as quantified via multilayer network analyses) correlates with social–communication symptom severity in ASD females (Gao et al. 2024), raising interpretative nuance around whether increased “integration” denotes an adaptive scaffold or constrained network rigidity. In parallel, structural connectivity-based studies in ASD females aged 12 to 30 years have revealed significant reductions in fractional anisotropy within white matter tracts underlying our morphology-based network profiles—including the internal capsule, inferior parietal lobule–lateral occipital cortex association fibers, and posterior corona radiata (Barnea-Goraly et al. 2010; Cheng et al. 2010). In sum, these convergent findings may suggest the presence of a multimodal (morphology, function, and structure) hub integration profile characterizing females with ASD.

Interestingly, when analyzing the full cohort, no significant sex-by-diagnosis interaction effects in morphology-based connectivity were observed, providing no evidence that diagnosis-related network alterations differed between males and females. This might suggest that such effect sizes were too small to be detected with our current sample size. We speculate that this could be partially attributed to integrating multiple morphological properties, which may capture shared network alterations more strongly than subtle sex-related differences.

Nevertheless, despite the lack of sex-by-diagnosis interaction effects, observed within-sex network alterations may help better understand ASD in females and males. To elaborate, since the fusiform gyrus appeared to be prominently hyperconnected in males, and given the established role of this brain area in face perception and social cognition (Kanwisher et al. 1997; Schultz et al. 2003; Schultz 2005), this could suggest a morphology-based substrate associated with compensatory mechanisms or atypical social attention pathways typically found in male-biased ASD studies. In females, prominent disruptions involved the entorhinal cortex, which may relate to differences in memory and spatial navigation in ASD relative to neurotypical controls (Coutureau and Di Scala 2009; Schultz et al. 2015). Importantly, the highlighted regions within each sex were not among those showing normative sex differences.

We assessed the sensitivity in discriminating utility of each morphological property underlying the obtained network profiles using a leave-one-feature-out procedure. Across sexes, the largest drops in diagnostic performance occurred when cortical thickness and sulcus depth were excluded, suggesting that these properties play a particularly sensitive role in the discriminative capacity of morphological networks—or at the very least, offer complementary information relative to the other morphological properties. Cortical thickness is a well-established marker known to be altered in autism and associated with symptom severity (Ecker et al. 2013; Yang et al. 2016; Khundrakpam et al. 2017; Lucibello et al. 2022). Sulcal depth, by contrast, has been less extensively studied in autism, with findings ranging from no statistical differences (Arutiunian et al. 2023), to specific localized abnormalities (Auzias et al. 2014), and to associations with early cortical folding processes during neurodevelopment (Nordahl et al. 2007). Our study builds on this body of work by highlighting the relevance of both of these morphological properties from a network-level perspective and across sexes. Conversely, removing either surface area or volumetric information from the morphology-based connectivity calculations led to slight improvements in discriminating utility, which seems to indicate a potentially redundant or less informative contribution from these properties. This can be partially explained by the strong correlation typically observed between surface area and volume at the cortex-level, so very little or none is gained from their combination. Nevertheless, in our analysis based solely on volumetric information, performed to allow the inclusion of the subcortex, we did not find any significant connected component of altered edges across sexes, reinforcing the limited utility of volumetric features to identify ASD individuals, at least from a network perspective. This appears to contradict prior studies suggesting that brain networks based on volumetric features have high predictive power for classifying ASD individuals (He et al. 2021). However, this may simply reflect the need for a more spatially distributed signature based on volumetric information, whereas our network-based statistical framework focuses on detecting clusters of significantly altered connections and might therefore be less efficient for classification purposes.

To assess whether network profiles can be related to a multivariate behavioral trait, we examined multivariate associations between morphology-based connectivity—restricted to those entries previously identified by NBS—and core autism-related behavioral measures within the autistic group. We found a significant brain–behavior association in males with autism, namely, a negative correlation with social and communication symptom severity, as provided by ADI-R subscores. Importantly, this result replicates recent reports using different approaches for estimating brain connectivity from multivariate morphology (Wang et al. 2024; Jiang et al. 2026), reinforcing the utility of morphology-based connectivity signals to capture aspects of behavioral heterogeneity in males with autism. On the other hand, no brain-behavior association was found in females, which is consistent with the possibility that their ASD-related behavior may be more multifaceted and arise from widespread network disruptions across the brain rather than mapping onto discrete and localized clusters of altered connectivity (Abbott et al. 2015; Benkarim et al. 2021; Buch et al. 2023). Finally, across both sexes, no statistical associations with comorbidity were found, suggesting that the observed morphology-based connectivity alterations are unlikely to manifest general traits of other psychiatric conditions.

Several limitations of our study should be acknowledged. First, the observed network profiles were restricted to individuals aged 8 to 18 years. Future studies should characterize similar profiles in adulthood, given the substantial changes in brain morphology that occur across the lifespan. Second, our data, drawn from Wave 1 of the ACE network, is cross-sectional and therefore precludes any conclusions about the developmental trajectory of our findings. However, subsequent waves (Wave 2 and 3) of the ACE network include follow-up data, which will enable us to test explicitly for longitudinal effects in future avenues. Third, our findings were derived using a purely statistical inference approach, which, although useful for gaining insight into the morphology-based network architecture characteristic of autism and its heterogeneity across sexes, does not quantify the utility of these features as biomarkers for diagnostic purposes. Future studies should adopt a thorough machine learning framework with out-of-sample testing to specifically assess this. Finally, our network results were obtained using a single neuroimaging modality, specifically, brain morphology. Hence, their combination with biomarkers from functional and structural connectivity signals may lead to a more comprehensive characterization of the ASD heterogeneity and improved diagnostic accuracy rates (He et al. 2025). Future studies should also examine the robustness and generalizability of network findings across modalities (Hull et al. 2017; Uddin et al. 2017).

Despite the aforementioned limitations, the present study provides evidence for the utility of brain connectivity signals based on integrative morphological information to further phenotype the neurobiology and behavior of males and females with autism. Accordingly, this contributes to a better understanding of heterogeneity in this neurodiverse condition across sexes and may provide new insights toward improved identification of autism in female individuals at younger ages, who are typically underdiagnosed.

## Supplementary Material

Supplementary_materials_bhag156

## Data Availability

Raw neuroimaging data used in this study can be downloaded from the National Institute of Mental Health Data Archive (collection #2021). Source files, analysis scripts and figures are available at https://github.com/RaseroLab/Autism-sex-stratified-morphology-connectivity.
