# Supplementary material for: Brain morphology network profiles in male and female youth with autism
Source: Cereb Cortex. 2026 Sep 28;36(9):bhag156. doi: 10.1093/cercor/bhag156 (PMC13617571; doi:10.1093/cercor/bhag156)
Supplement: Supplementary file 1 [file supplementary_materials_bhag156.pdf]

Supplementary Materials

Brain morphology network profiles in male and female youth with autism

Nooshin Safari[1], Isaac Levy[1], Aiying Zhang[1], Chirag Agarwal[1], Jesus M. Cortes[2,3,4], Kevin A. Pelphrey[5], John Darrell Van Horn[1,6], Javier Rasero*[1]

[1]School of Data Science, University of Virginia, Charlottesville, VA, USA.

[2]Computational Neuroimaging Laboratory, BioBizkaia Health Research Institute, Barakaldo, Bizkaia, Spain.

[3]Ikerbasque, Basque Foundation for Science, Bilbao, Spain.

[4]Department of Cell Biology and Histology, University of the

Basque Country (UPV/EHU), Leioa, Bizkaia, Spain.

[5]Department of Neurology, University of Virginia, Charlottesville, VA, USA.

[6]Department of Psychology, University of Virginia, Charlottesville, VA, USA.

*Corresponding author:

Javier Rasero

School of Data Science, 1919 Ivy Rd, Charlottesville, VA 22903, United States of America.

mnr3kn@virginia.edu

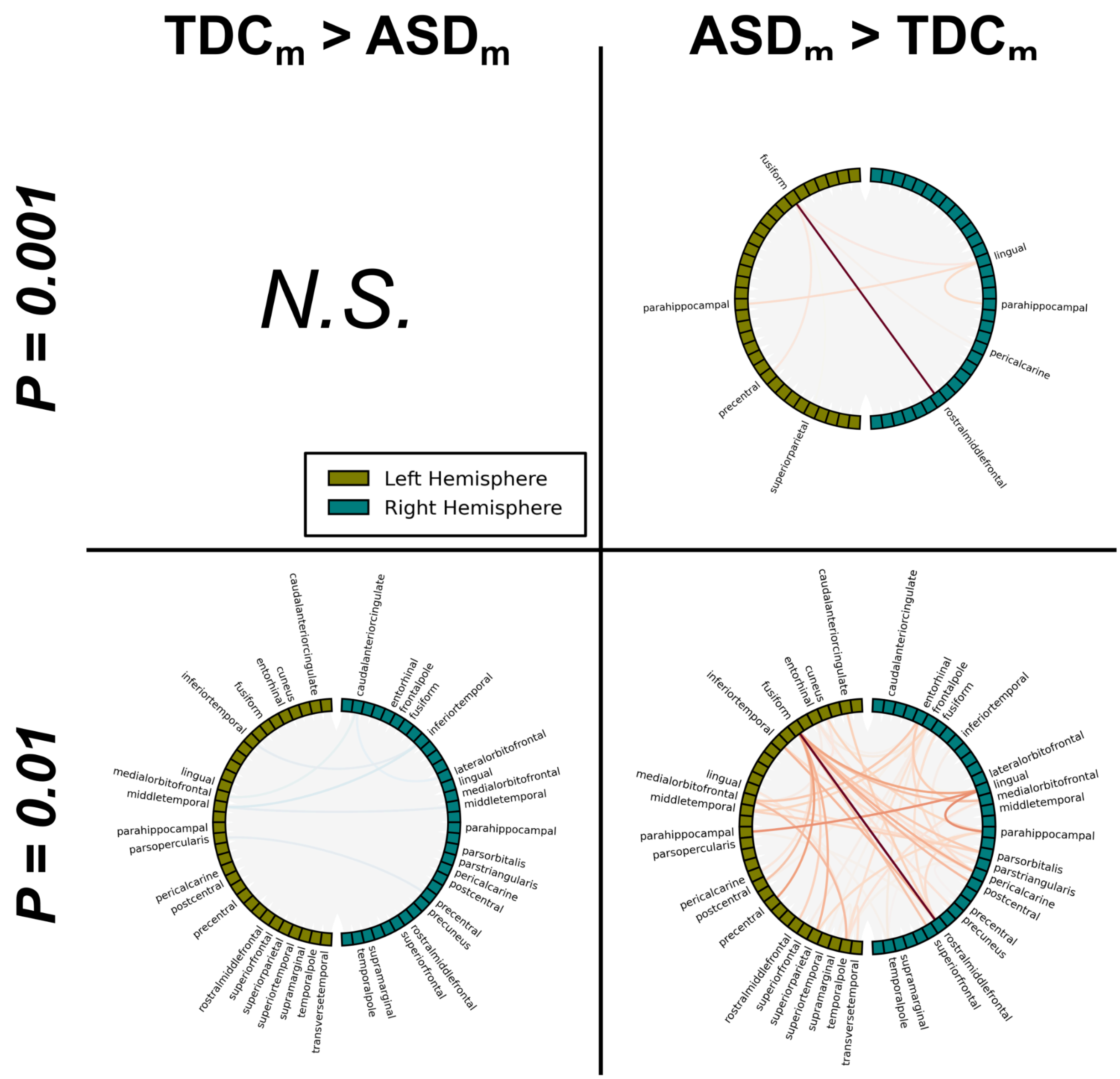

Fig S1: ***Network profiles with different individual edge thresholds (male cohort)****. Circular plots visualizing clusters of significant inter-regional connections using a more stringent individual edge threshold ($p = 0.001$, first row) and a more lenient threshold ($p = 0.01$, second row), than in the main analysis ($p = 0.005$). Nodes represent cortical regions, with olive and teal indicating the left and right hemispheres, respectively. Blue colors indicate increased morphology-based connectivity in the TDC group relative to ASD (left panels), and red colors the opposite (right panels).*

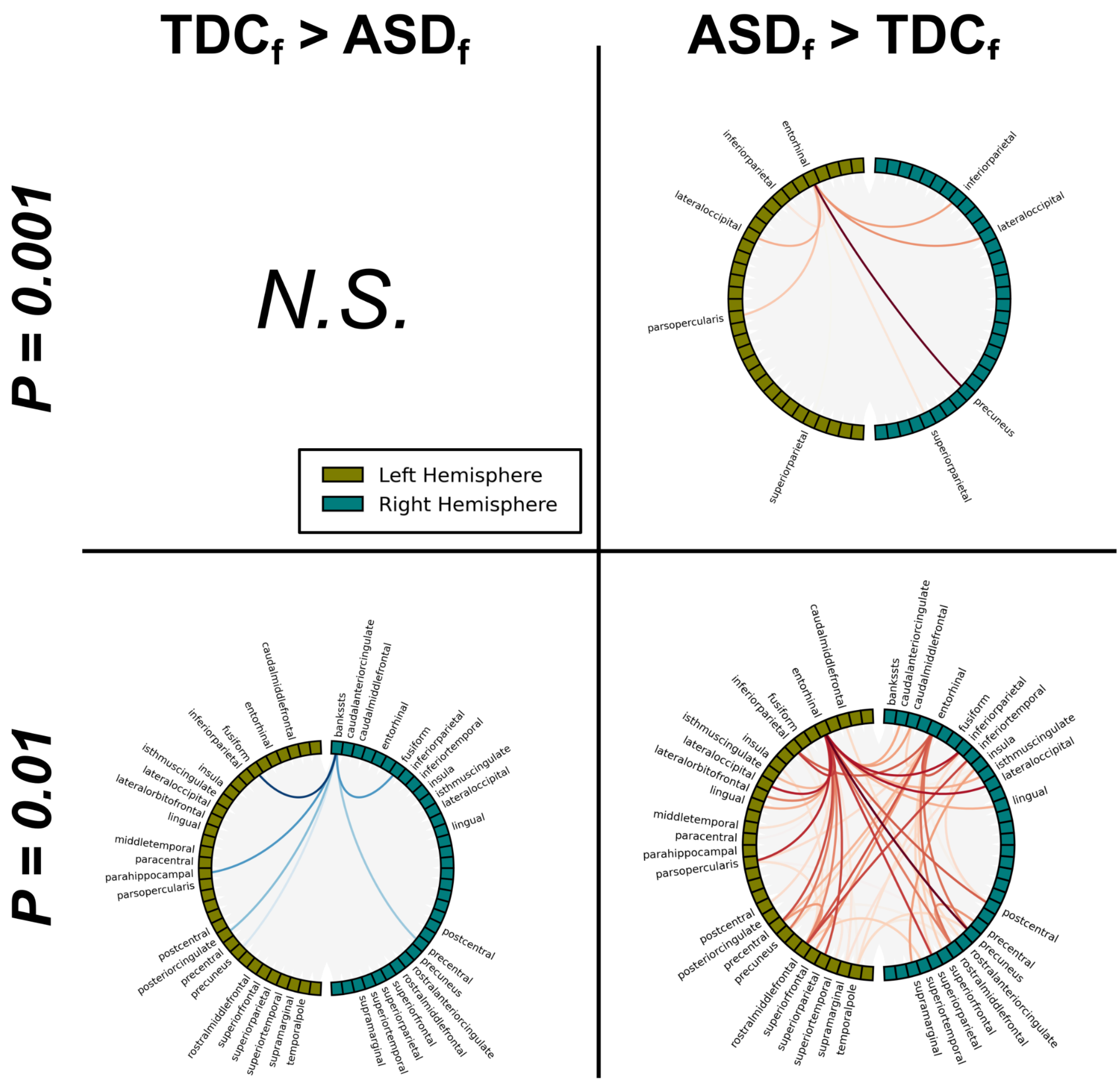

Fig S2: ***Network profiles with different individual edge thresholds (female cohort)****. Circular plots visualizing clusters of significant inter-regional connections using a more stringent individual edge threshold ($p = 0.001$, first row) and a more lenient threshold ($p = 0.01$, second row), than in the main analysis ($p = 0.005$). Nodes represent cortical regions, with olive and teal indicating the left and right hemispheres, respectively. Blue colors indicate increased morphology-based connectivity in the TDC group relative to ASD (left panels), and red colors the opposite (right panels).*

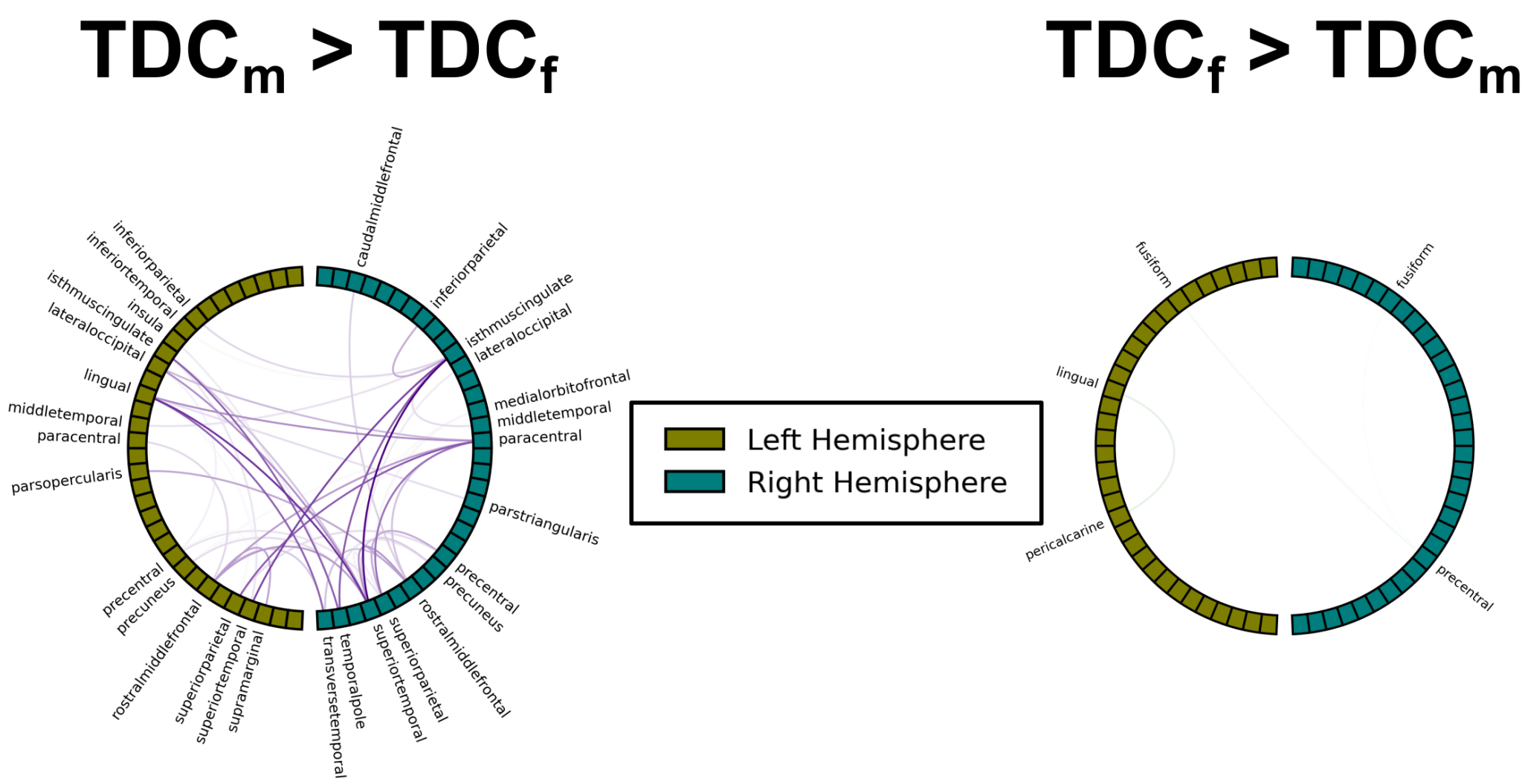

Fig S3: ***Sex-normative morphological network profiles****. Circular plots visualizing clusters of significant inter-regional connections comparing males and females within the TDC group. The same setup as in the main analysis was adopted (individual edge threshold p = 0.005; FWE-corrected p = 0.05; 5,000 permutations). Nodes represent cortical regions, with olive and teal indicating the left and right hemispheres, respectively. Indigo colors indicate increased morphology-based connectivity in males relative to females, and green the opposite.*
